# Editorial: Rising stars in chronobiology 2022

**DOI:** 10.3389/fphys.2024.1412956

**Published:** 2024-04-25

**Authors:** Joanna C. Chiu

**Affiliations:** Department of Entomology and Nematology, College of Agricultural and Environmental Sciences, University of California, Davis, CA, United States

**Keywords:** circadian rhythm, sleep loss, gene expression, adipose tissue, high-fat diet, diet-induced obesity, tumor, metabolism

Chronobiology is the study of biological rhythms. The majority of studies in chronobiology to date have largely focused on circadian or 24-h rhythms, but there have been increasing interests in non-circadian biological rhythms, including seasonal rhythms (Wood and Loudon, 2014; Liams et al., 2019; Rohr et al., 2019; Abrieux et al., 2020) and circatidal rhythms (Kwiatkowski et al., 2023; Lin et al., 2023). The understanding of circadian rhythm has come a long way since Jean-Jacques d’Ortous de Mairan’s 1729 observations that the daily rhythm of leaf movement in *Mimosa pudica* plants persisted even when plants were placed in the dark (de Mairan 1729). His observations provided the first evidence to support the endogenous nature of circadian rhythms. Early evidence illustrating the genetic basis of circadian rhythm were documented centuries later when Bünning showed that crosses of *Phaseolus* bean plants with different period lengths with regard to leaf movement produced hybrid plants with intermediate period lengths, suggesting that circadian period length of leaf movement is an inherited trait (Bünning, 1932). An excellent account of the history of chronobiology has been compiled by the Society for Research on Biological Rhythms (SRBR) (https://srbr.org/about-chronobiology/chronohistory/).

The scope of circadian biology is immense and the impact of circadian rhythm is pervasive. Despite all the advances in the field in the past century, there is still much to discover. Given organisms from all domains of life inhabiting a range of ecological niche are found to possess circadian clocks, and cutting-edge genomic editing tools and high throughput genomic technologies now enable researchers to study rhythms in non-model species in more natural conditions, there are now increasing opportunities to study circadian rhythms that are unique and critical to understanding species-specific biology, e.g., swarming behavior in mosquitoes (Wang et al., 2021), social entrainment in colony insects (Siehler et al., 2021), and activity rhythms in Cnidarians (Kanaya et al., 2019). Conversely, the use of diverse models in the field of chronobiology will enable comparative analysis and formulation of broad principles governing circadian rhythm and its adaptive nature. Finally, the impact of chronobiology on biomedical sciences is hard to ignore, especially after 2017 when pioneering chronobiologists Michael Young, Michael Rosbash, and Jeffrey Hall, were awarded the Nobel Prize in Physiology and Medicine “for their discoveries of molecular mechanisms controlling the circadian rhythm.” In addition to established links between circadian disruption and many human diseases (reviewed in Manoogian and Panda, 2017; Masri and Sassone-Corsi, 2018; Acosta-Rodriguez et al., 2021; Lopez-Otin and Kroemer, 2021), chronobiologists and clinicians are increasing recognizing the potential of incorporating circadian medicine into healthcare and medical treatments (Ruben et al., 2019; Rasmussen et al., 2022; Kramer, 2023; Sole and Martino, 2023; Mangini et al., 2024; Zhu et al., 2024).

The field of chronobiology is growing rapidly; pioneering scientists have built a strong foundation and new investigators are infusing innovative ideas and creativity to accelerate new discoveries in basic principles of chronobiology while others are working towards leveraging circadian biology for precision medicine. The “*Rising Stars in Chronobiology*” Special Research Topic features the research of some of the up-and-coming investigators in the field of chronobiology. In Xin et al., Min-Dian Li and his group took a systems approach to define the circadian signatures in the adipose tissue of diet-induced obese mice and the impact of dietary intervention. Although the impact of malnutrition on liver circadian reprograming has been extensively studied (Eckel-Mahan et al., 2013; Guan et al., 2018; Martini et al., 2023), its impact on other peripheral tissues, e.g., adipose tissue, is less clear. Given different organs may respond to nutritional cues in distinct manner, this study provides new insights into how high fat diet can differentially alter circadian rhythms in different organ systems, potentially disrupting overall circadian synchrony.

In Verlande et al., Selma Masri and her group, who are at the forefront of circadian and cancer biology, demonstrated that a stable isotope ^13^C-glucose tracer, once injected in animals, can be monitored in real-time in exhaled breath to quantify glucose metabolism. They further validated this approach in models of diet-induced obesity and lung tumor progression, in which metabolic dysregulation is expected. Given circadian disruption is associated with metabolic dysregulation, this stable isotope technology will be valuable for studies in chronobiology.

In Wang et al., Wanhe Li collaborated with Nobel Laureate Michael Young to leverage *Drosophila* sleep mutants and investigate the impact of chronic sleep loss on rhythmic gene expression. They observed significant dampening in rhythmic gene expression in sleep mutants when compared to wild type flies. Interestingly, they found a subset of genes whose rhythmic gene expression was dampened in old flies and sleep mutants with chronic sleep loss, suggesting that aging may contribute to sleep loss induced disruption of circadian gene expression.

In Yuan et al., Swathi Yadlapalli and her group lamented that although subcellular localization of core clock proteins, such as CLOCK, are known to play a critical role in circadian clock function (reviewed in Patke et al., 2020), whether and how the spatiotemporal organization of clock mRNAs regulate circadian clock function have not been established. To address this question, they developed a streamlined single molecule fluorescence *in situ* hybridization (smFISH) protocol in combination with an automated machine-learning-based data analysis pipeline to quantify and assess localization of key clock mRNAs. They presented successful validation of their method by quantifying spatiotemporal expression of *clock* mRNA in *Drosophila* clock neurons.

Finally, in Anna et al., Nisha Kannan and her group leveraged the genetic tools in the *Drosophila* fly model to investigate the role of post-transcriptional mechanisms in regulating circadian rhythms. In particular, they characterized the role of miRNA-277 (miR-277) by downregulating its expression in multiple groups of clock neurons in the fly brain. They concluded that miR-277 plays a role in fine tuning the expression of *Clk* mRNA and in maintaining robust circadian rhythms.

As I am concluding this editorial, I would be remiss if I do not acknowledge the significant contribution of my late colleague Steve Brown to this Special Research Topic. I started this editorial assignment as a co-editor with Steve, who as many in the field of chronobiology know, was not only a brilliant scientist (Gachon, 2024), but a fierce supporter and mentor of junior colleagues and trainees. Steve was very excited to have the opportunity to highlight research by rising stars in the field. Tragically, Steve passed away in a plane crash on 14 December 2022. He now lived on in the memories of his family, friends, and others; he will be remembered by his colleagues in chronobiology for his joy for life and adventures, his innovation and scientific brilliance (e.g., Brown et al., 2005a; Brown et al., 2005b; Dallmann et al., 2012; Azzi et al., 2014; Gaspar et al., 2017; Brüning et al., 2019; Noya et al., 2019; Collins et al., 2020), and his generosity and kindness as a colleague, friend, and mentor.
